# The Effect of Low Frequency Sound on Heart Rate Variability and Subjective Perception: A Randomized Crossover Study

**DOI:** 10.3390/healthcare10061024

**Published:** 2022-06-01

**Authors:** Zdeněk Vilímek, Jiří Kantor, Jakub Krejčí, Zbyněk Janečka, Zuzana Jedličková, Anna Nekardová, Michal Botek, Monika Bucharová, Elsa A. Campbell

**Affiliations:** 1 Center of Evidence-Based Education and Arts Therapies: A JBI Affiliated Group, Institute of Special Education, Sciences, Faculty of Education, Palacký University Olomouc, 779 00 Olomouc, Czech Republic; jiri.kantor@upol.cz (J.K.); or andulkanekardova@seznam.cz (A.N.); monika.bucharova01@upol.cz (M.B.); elsa.campbell@caritas-ettlingen.de (E.A.C.); 2 Faculty of Physical Culture, Palacký University Olomouc, 771 11 Olomouc, Czech Republic; jakub.krejci@upol.cz (J.K.); zbynek.janecka@upol.cz (Z.J.); zuzana.jedlickova01@upol.cz (Z.J.); michal.botek@upol.cz (M.B.); 3 Caritas Association Ettlingen, 76275 Ettlingen, Germany

**Keywords:** vibroacoustic therapy, low frequency vibration, nature sound, heart rate variability, subjective perception, cardiac autonomic regulation, stress, mood

## Abstract

Background: Vibroacoustic therapy (VAT) uses low-frequency sound, often combined with listening to music, for therapeutic purposes. However, the impact of low-frequency vibration (LFV) on physiological functions and subjective perception is relatively unknown. Methods: We conducted a randomized cross-over study with the aim of comparing the effect of constant LFV of 40 Hz, its amplitude modulation, and the placebo condition on heart rate variability (HRV), stress perception (measured by visual analogue scales for stress) and mood (measured by UWIST Mood Adjective Check List). Results: Research experiments with various interventions (constant LFV with sound of nature (river in forest), amplitude modulation of the same LFV with sounds of nature and sounds of nature without LFV) were realised involving 24 participants. It was found there was an effect on HRV, stress perception and mood after the interventions. However, there were only seldomly experienced, and mostly nonsignificant, differences between the intervention conditions, so the effects may be attributed to factors other than LFV. Conclusions: Large scale experimental studies are needed to verify the preliminary findings and to explore various coinciding factors that may have influenced the results of this study, e.g., type of autonomic nervous system. We propose that the effect of LFV exposure may differ when combined with listening to music, and this hypothesis should be investigated in future studies.

## 1. Introduction

Vibroacoustic therapy (VAT) is defined as a combination of low-frequency sound vibration, (and) listening to music, combined with therapeutic interaction [1]; however, there is great heterogeneity in the range of frequencies, usage of music and applying the low frequency vibrations (LFVs) within a therapeutic relationship. This therapeutic approach incorporates physical and auditory perception of both auditory sound and tactile vibration. In research studies, the therapeutic relationship has been less discussed and, therefore, the focus of the research has been on the tactile stimulus itself, this being the low frequency vibration. There is also a tendency to research the effects of LFV without the addition of the listening to music element that enables differentiating of the effect of music from the effect of LFV [2]. Although this may lead to greater understanding of the standalone effects of LFV, knowledge on these combined elements is lacking. Since anecdotal evidence from clinical practice, as well as from some case reports, e.g., Campbell et al. [3], has shown that some find LFV alone emotionally or physically stressful, music is often added to support the psychological processing and because it may enhance the relaxation effect. Therefore, more evidence is needed on the combined effects of LFV and music. 

Vibroacoustic therapy can be easily altered to meet the specific needs and therapeutic goals of the individual. This is enabled through adjusting different parameters of LFV treatment programmes in various systems, such as Nextwave or VibLab. The frequencies (Hz) used are based on years of clinical experience [4] and neuroscientific principles, such as oscillation coherence [5]. In recent research, 40 Hz, either as a stand-alone frequency or as the fundamental frequency within a treatment programme, has been used most commonly, as it induces a whole-body relaxation response [3,6,7,8]. Amplitude refers to the loudness peak from silence to the designated crest of a frequency. In the Nextwave system, the pulsation or cycle refers to the speed of the amplitude change; the duration from silence to the designated peak volume (in decibels). The strength/loudness is the intensity of the stimulation, which can be altered according to each speaker or transducer in the device. Finally, action or direction refers to whether the vibration moves from head to foot, vice versa or remains fixed [9].

Years of clinical experience [10], as well as some research data, indicate VAT may be used for stimulation of physiological functions, e.g., heart rate variability (HRV) [11,12,13], which is the non-invasive index of autonomic cardiac regulation. Other physiological functions sensitive to VAT include galvanic skin response [12], blood pressure [14,15,16], or spasticity and movement [17,18]. Some studies report the effect of VAT on subjective perception of stress [19,20,21]. There is a generally accepted hypothesis that VAT increases the reactivity of cardiac vagal regulation and physical relaxation [12,22]. However, there is a lack of research in this area. Most of the research cited was carried out in a traditional manner, combining music with LFV. Until now, we do not know much about the effect of LFV when unaccompanied by musical intervention. There is also little research focused on the comparison of different parameters of LFV, such as frequencies or amplitude modulation. 

The aim of this research study was to compare the effect of two types of sinusoidal signals on heart rate variability and subjective perception of stress: constant frequency of 40 Hz and amplitude modulation of this frequency. Our hypothesis is that VAT will stimulate increased cardiac vagal regulation and a feeling of subjective physical relaxation. We used two types of vibroacoustic beds available at the Palacký University in Olomouc (see Section 2.1) to generate the two different types of LFV and to compare the intervention by constant (Carrier signal or wave) amplitudes of 40 Hz (generated on VIBRATICS I) with the intervention of sequentially dosed amplitudes (generated on Vibrobed). We proposed that VAT should have significant effect on the increase of cardiac vagal activity and decrease blood pressure, and that this physiological effect should be reflected in decrease of stress perception in participants.

## 2. Materials and Methods

The design of pre-test post-test randomized cross-over study with three interventions in randomized order was used to:

- Determine the effect of constant LFV with sounds of nature (sounds of river in the forest) on heart rate variability.
- Determine the effect of LFV with nature sounds on subjective perception of acute stress.
- Determine the effect of LFV with nature sounds on participants’ mood.

This pilot study was conducted to prepare for a RCT study with a large group of participants. The study was approved by the ethics committee of the Faculty of Education, Palacký University in Olomouc (No. 5/2019). All participants were informed in advance of the conditions of the research experiment, signed an informed consent (Appendix A) and filled in the entry protocol (Appendix B). The sample of participants was created from full-time students of the Faculty of Education and the Faculty of Physical Culture of Palacký University in Olomouc. The assumption was that these students would be a sufficiently homogeneous group to verify the assumptions, due to their small age range and similar lifestyles. In addition, this sample can potentially benefit from the positive effects of VAT due to the increased presence of acute stress [23,24]. However, in this study we recruited students with any level of stress (see Strengths and limits of the study in Section 4). When selecting study participants, we first addressed several groups of students from the Faculty of Education and the Faculty of Physical Culture by e-mail. All students who agreed to participate in the study, and met the inclusion criteria, were included in the study. All participants received basic information about the goals and methods of conducting the research survey and signed informed consents before the first meeting. Data collection took place from April 2019 to June 2019. To ensure the anonymity of participants, all data were traceable only under special codes, based on acronyms composed of letters and numbers that were generated by a computer random number generator.

The inclusion criteria were as follows:

- Voluntary participation in the study
- Czech or Slovak nationality
- Full-time students aged 18–30 years

The exclusion criteria were the following:

- Psychiatric diagnosis, neurologic disease (e.g., epilepsy, cerebral palsy) or any known contraindication of VAT, such as muscle hypotonia, angina pectoris, psychotic or acute post-traumatic conditions and bleeding [16]
- Post-traumatic stress disorder or perceived pain
- Sleep deprivation
- Substance abuse (caffeine, nicotine) on the day of the experiment or, in the case of strong addictive substances (alcohol), one day before the experiment
- Vigorous physical activity one day before experiment
- Menstruation
- Consumption of food two hours before the experiment
- Medication or dietary supplements affecting the autonomous nervous system
- (Self-reported) cardiovascular or metabolic conditions.

### 2.1. The Description of the Research Experiment

**Figure 1:**
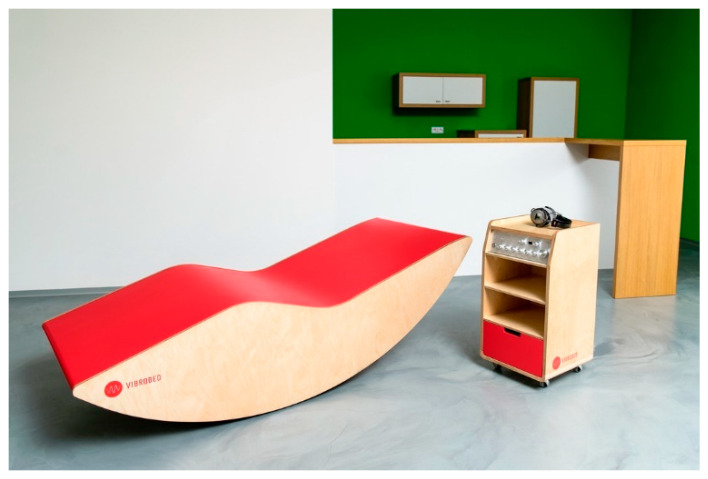
Rehabilitation vibration bed VIBROBED^®^ (from the authors’ archive).

We used two different VAT devices that have been developed in the Czech Republic in recent years: the Rehabilitation vibration bed VIBROBED^®^ and Vibrating lounger VIBRATICS I. VIBROBED^®^ (Figure 1) is a rehabilitation vibration bed, constructed by Czech designers Zdeněk Vilímek and Petr Švarc in 2016. One of the unique features of the Vibrobed is close interaction between the low-frequency sound and the music listened to by the client; these are synchronously mixed to form a complete unified audio composition and transmitted to four zones of the body schema (calves, thighs, upper back area, and low back area). The Vibrobed has its own four-channel amplifier, music player and low-frequency sound generator with various sinusoidal signal settings that can be selected according to clients’ individual needs. 

An amplifier of 4 × 100 W/2 Ohm with a built-in module composed of audio player and generator was used; the modulation and modulated signal are sinewave. The output of the amplifiers leads to vibrating exciters (electrodynamic transducers) and to headphones (without a modulated signal of generator). Several potentiometers are used for independent control of vibration levels in each zone and a rotary encoder to control functions of the generator/player. Nominal impedance of one electrodynamic transducer is 4 Ω and power capacity is 50 W RMS. Four body zones (Z1 = calf area 2×, Z2 = thigh area 2×, Z3 = cross area 2×, Z4 = upper back area 2×) are excited by four pairs of transducers (one zone contains a pair of closely placed transducers connected in parallel, with total nominal impedance of 2 Ω per zone) fixed to a resonant membrane (special wood composite). A total of 8 vibration exciters are placed under the body scheme. Their exact location, composition of the resonant membrane, acoustic and software modifications, etc. are the subject of the author’s solution. 

The innovative Vibrobed technology uses primary sequential dosing of LFV (amplitude modulation with different time courses, sine sweep up and down in the frequency range 30–80 Hz) and various modulations of low-frequency waves in combination with sound and music stimuli. 

**Figure 2:**
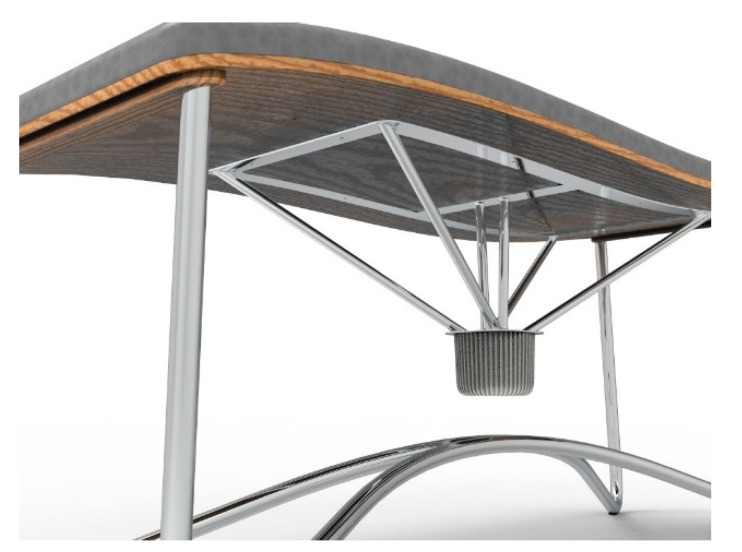
Vibrating lounger VIBRATICS I (from the authors’ archive).

VIBRATICS I is a vibrating bed with wooden board and with a softened hygienic surface (Figure 2). Transmission of vibrations is generated by the quiet subwoofer Buttkicker LFE, which has extremely low bass frequencies from 5 to 200 Hz, through a set of rods that are attached to the bottom of the lounger. The frequency modulated continuous sine signal is provided by the Rohde and Schwarz function generator. Power Handling is 400 W, nominal impedance is 4 Ω. The frequency of the signal used in the experiment was 40 Hz.

**Figure A1:**
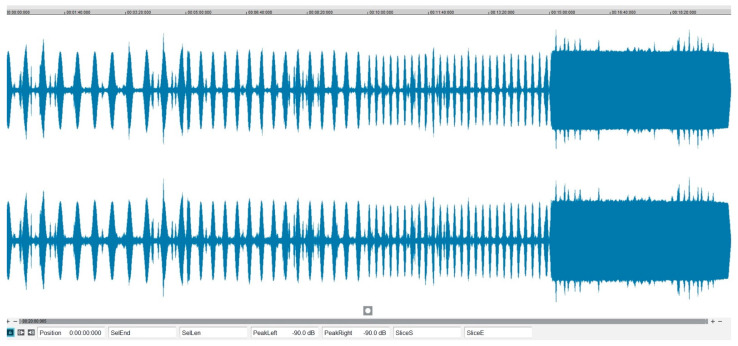
Visualisation of Amplitude Modulation used by Vibrobed.

The course of the experiment was the same in all cases, differing only in the type of intervention provided. Each participant attended a total of three meetings, once a week, always in the morning on the same day of the week, at the same time. Individual meetings included:

- 20 min. stimulation with constant sinusoidal wave 40 Hz (carrier signal) LFV without modulation (generated on VIBRATICS I), accompanied by nature sounds (recording of river sounds in forest), in supine position (experimental intervention 1).
- 20 min. stimulation with specific modulation of LFV (40 Hz) (generated on Vibrobed) accompanied by nature sounds of river in forest, in supine position (experimental intervention 2). The detailed description of the amplitudes is below and in Appendix C (Figure A1 and Table A1).
- 20 min. without any LFV, supine position (control intervention), accompanied by nature sounds (recording of river sounds in forest). Measured on both types of vibroacoustic beds (half of the participants measured on Vibrobed, the other half measured on VIBRATICS I).

Constant (carrier signal) LFV is a traditionally used type of low-frequency stimulation, while amplitude modulation of LFV, with different durations, has not yet been sufficiently studied. In the case of both types of LFV, the same frequency of 40 Hz, comparable strength of low-frequency waves and the same type of recording of natural sounds (river sounds in forest) were used. Concerning the amplitude modulation, three types of amplitude modulation (AM) 40 Hz signal with different cycle lengths and then 5 min pure unmodulated 40 Hz signal (carrier signal), provided a total length of stimulation of 20 min. The contrasting effect of the sequentially dosed series was then considered and involved gradually shortening the cycle length (14 s, 10 s, 6 s = from long to short) to the original shape of the 40 Hz carrier signal. Each AM cycle was supplemented by silence at the same length as the length of one modulation signal cycle (stimulus processing time and organism response):

- Sequence 1: alternating AM 1 (14 s) + silence (14 s), length 5 min.
- Sequence 2: alternating AM 1 (10 s) + silence (10 s), length 5 min.
- Sequence 3: alternating AM 1 (6 s) + silence (6 s), length 5 min.
- Sequence 4: pure unmodulated signal 40 Hz (Carrier Signal or Carrier Wave), length 5 min.

It is visualized in Appendix C. The *modulation* index (or *modulation depth*) of amplitudes was 70–75%.

Non-commercial recordings of nature sounds (sounds of river) from recording Studio Reset (Brno, Czech Republic) were used. Participants listened to the natural sound with earphones, and loudness was set up according to participants’ preferences. Participants did not rate their preference or experience of nature sounds. The control intervention took place in a supine position under similar conditions. The order of the three interventions was randomized using a randomization table generated before starting the whole experiment with a random number generator (the randperm function available in MATLAB R2018b, MathWorks, Natick, MA, USA). This was a single-blind study; the participants did not know their order of intervention while, for logistical reasons, the researchers knew the order of intervention.

### 2.2. Outcome Measures

Outcome measures included frequency indices of heart rate variability, Visual analogue scales (VASs) for stress, UWIST Mood Adjective Check List, Perceived Stress Scale-10 (Czech version) and a questionnaire for personal and demographic data.

**Figure 3:**
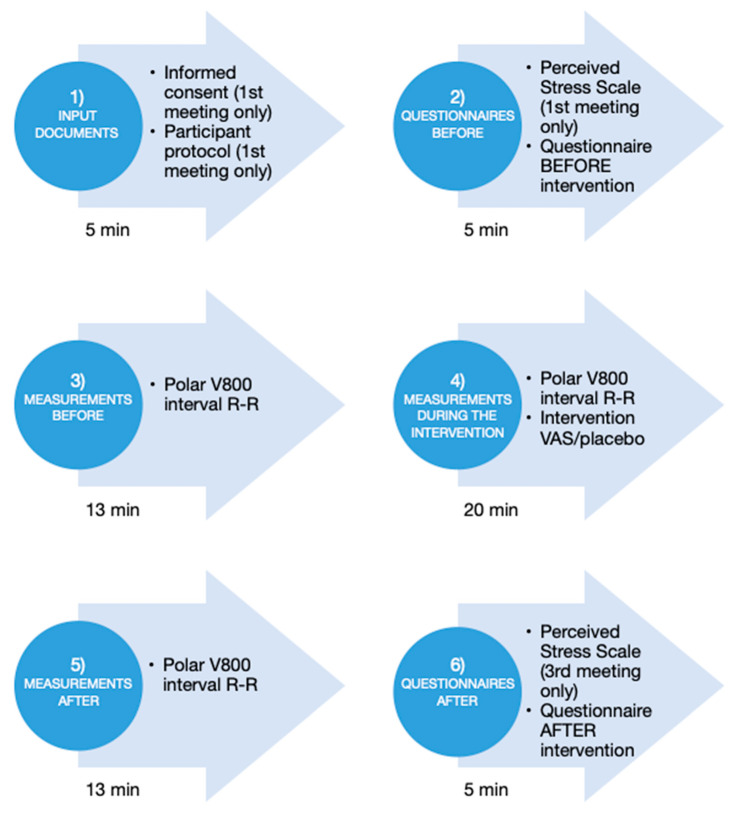
Procedure of the research experiment with outcome measures used (VAS-S and UWIST-MACL were included in the questionnaire before/after the intervention).

In this study, HRV frequency indices were used as the primary physiological outcome. HRV analysis provides a non-invasive method for evaluating cardiac autonomic regulation [25]. To calculate HRV frequency indices, the electrocardiogram was recorded using a Polar V800 heart rate monitor (Polar, Kempele, Finland), which has been proved to be a valid device [26]. RR intervals, which were defined as the duration between two adjacent heartbeats, or, more precisely, as the duration between R waves on the electrocardiogram, were measured with a resolution of 1 ms. HRV was analyzed before and after the interventions by means of orthoclinostatic maneuver (Figure 3). The orthoclinostatic maneuver lasted 12 min and consisted of 5 min standing and 7 min in a supine position (with a 1 min stabilization period) [27]. HRV was also continuously analyzed during the intervention lasting 20 min [28]. The RR intervals were visually examined by an expert, and all artifacts were manually filtered. From the orthoclinostatic maneuver, two 5-min segments (standing, supine) were extracted. From the recording taken during the intervention, four adjacent 5 min segments were extracted. HRV frequency indices were calculated using a special application DiANS PF8 (DIMEA Group, Olomouc, Czech Republic) from measured RR intervals. The calculations were based on spectral analysis performed using Fast Fourier Transform with a sliding 256 points Hanning window. The power spectra were quantified by integrating the area under the power spectral density curve. Two frequency bands were used: low frequency (LF) from 0.05 to 0.15 Hz, and high frequency (HF) from 0.15 to 0.50 Hz. The HF power was solely modulated by cardiac vagal activity, while the LF power was associated with baroreflex and sympathetic activity on the sinus node, and the LF/HF ratio reflected sympathovagal balance [21]. The respiration activity was not monitored.

Visual analogue scale (VAS) for stress [29] involves a standardized blank line, 10 cm long, anchored by the extreme values of 0 and 10. On the scale, participants recorded the degree of acute stress experienced (0 = no stress, 10 = maximum stress) before and after the intervention.

The UWIST (University of Wales Institute of Science and Technology) Mood Adjective Checklist (UWIST-MACL) is a 24-item adjective checklist assessing the moods or feelings that people have. The participants could score their mood on a 1–4 scale, ranging from Definitely (1) to Definitely not (4) [30]. Score 1 means the participant is definitely feeling the mood in the moment, e.g., happy, energetic or nervous. We included this questionnaire as an additional measure, because it had been used in Wigram’s work on vibroacoustic therapy [16] and it would help us to determine the effect of VAT on the participants’ subjective perceptions. It was applied as a pre-test post-test measurement. 

The Perceived Stress Scale-10 [31], the Czech version [32], is a standardized self-assessment scale for measuring acute perceived stress. PSS-10 was measured before the first, and after the third, intervention to determine whether the stress level changed significantly during the research experiment due to external influences or not. This questionnaire consists of 10 items related to the subjective evaluation of stressful situations during the last month. Participants answered by choosing from the following options: never, almost never, sometimes, quite often and very often, and were assigned points 0-4. The results were evaluated using the arithmetic mean of all items. All recruited participants were included into the study, notwithstanding the initial level of stress measured by PSS-10 and VAS-stress.

### 2.3. Statistical Analysis

All data were imported into Excel 365 (Microsoft, Redmond, WA, USA) tables for descriptive statistical processing. Data were presented as arithmetic mean and standard deviation. Frequency indices (LF, HF, LF/HF) were transformed using the natural logarithm (Ln) to achieve a normal distribution. The normality of the transformed data was verified using the Kolmogorov-Smirnov test. Analysis of variance (ANOVA) for repeated measures, with two factors (intervention factor and time factor) and interaction, was used to analyse the heart rate and frequency indices. The intervention factor has the following three levels: constant amplitude (Con), amplitude modulation (Seq), and placebo intervention (Pla). The time factor has two levels for orthoclinostatic maneuver (Pre and Post intervention) or four levels for the analysis of the intervention record (1–5 min, 6–10 min, 11–15 min, and 16–20 min). To increase statistical power, the four values obtained during the intervention were pooled and evaluated using the planned contrast [33].

All remaining variables (perceived stress, and UWIST items) were compared using the paired Wilcoxon test. A nonparametric test was preferred because some statistical variables differed significantly from the normal distribution. No adjustment was used in this study to control for Type 1 statistical error. For all statistical tests, *p* < 0.05 was considered statistically significant. Statistical analyses were performed using MATLAB with Statistics Toolbox R2020a (MathWorks, Natick, MA, USA).

## 3. Results

Out of the total number of 24 participants, two participants did not complete all measurements because of illness (data from these two participants were not included in the data analysis). The group consisted of 12 men (55%) and 10 women (45%). Participants were aged 27.3 ± 11.9 years (mean ± standard deviation). None of the participants was in treatment due to a psychiatric or neurological illness, and none of the participants had a disability or a severe acute illness. Regular and sufficient sleep at the time of the experiment was reported by 20 participants (91%). The average length of sleep before the research experiment was 7.01 (range 4–8.5) hours. The presence of menstruation and the menstrual cycle was also recorded for female participants, with 5 women (50%) reporting a follicular phase and 5 women (50%) reporting a luteal phase. The sums of the participants’ total scores, regarding perceived stress (evaluated by PSS-10), were 20.9 ± 2.0 at the beginning of the research experiment and 20.1 ± 1.9 at the end of the experiment. Thus, there was no statistically significant change (*p* = 0.084) in the participants’ stress load during the experiment; in this case, we would evaluate the stress level as a medium load.

### 3.1. The Effect of Low-Frequency Sound on the Heart Rate Variability in Pre Test/Post Test Measures

**Figure 4:**
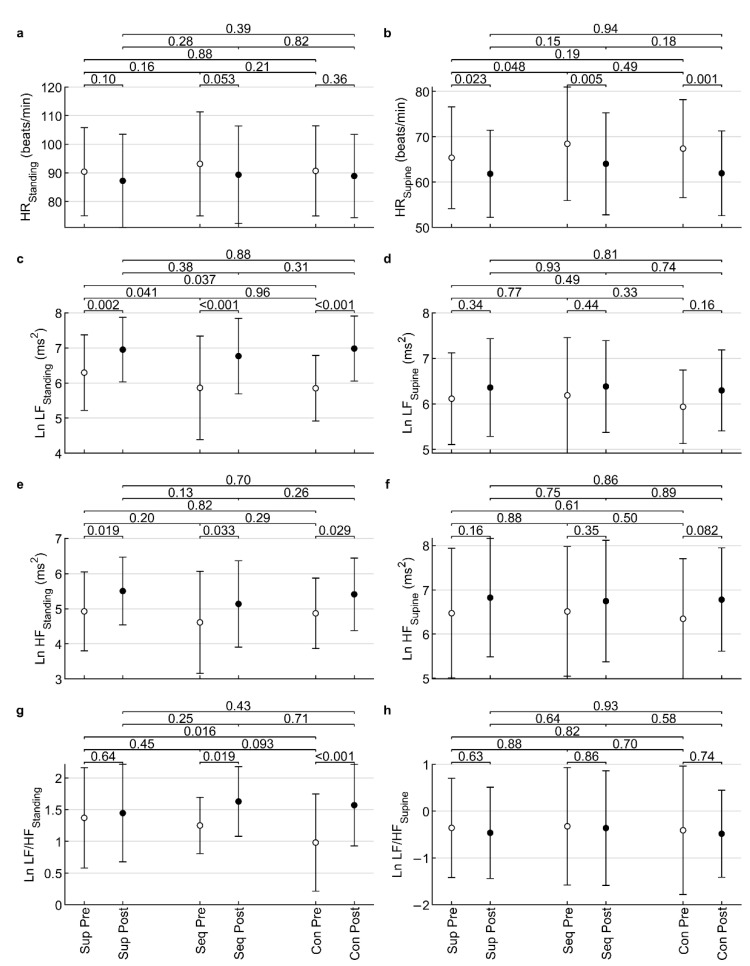
Influence of low-frequency sound on heart rate (**a**,**b**); low-frequency power (**c**,**d**); high-frequency power (**e**,**f**); and low-frequency/high-frequency ratio (**g**,**h**) in pre/post measures. Standing—standing body position in the orthoclinostatic maneuver; Supine—supine body position in the orthoclinostatic maneuver; Ln—natural logarithm; Sup—placebo supine; Seq—stimulation with amplitude modulation of LFV; Con—constant LFV. The values displayed above the clips are *p*-values of Fisher’s LSD tests.

The Kolmogorov-Smirnov test showed that the indices HR_Standing_ (*p* = 0.037), HR_Supine_ (*p* = 0.004), Ln HF_Standing_ (*p* = 0.006), and Ln LF/HF_Supine_ (*p* = 0.021) differed significantly from the normal distribution. The remaining indices did not differ significantly (all *p* ≥ 0.10) from the normal distribution. After visual inspection of the histograms, the deviations from normality were found to be small and ANOVA was considered robust enough for such deviations from normality [26]. For this reason, ANOVA could be used, despite it requiring the assumption of data normality. The results of the ANOVA are shown in Table 1 and further explored in Figure 4.

ANOVA revealed a statistically significant time factor (all *p* ≤ 0.019) for HR_Standing_, HR_Supine_, Ln LF_Standing_, Ln HF_Standing_, Ln HF_Supine_, and Ln LF/HF _Standing_, suggesting that interventions affected HRV indices in the post-test compared to the pre-test. However, no statistically significant intervention factor (all *p* ≥ 0.054) or interaction (all *p* ≥ 0.076) was found for any HRV index. Therefore, it was not possible to find a difference in the effect after the studied interventions in this study.

### 3.2. The Effect of Low-Frequency Sound on Hear Rate Variability during Intervention

**Figure 5:**
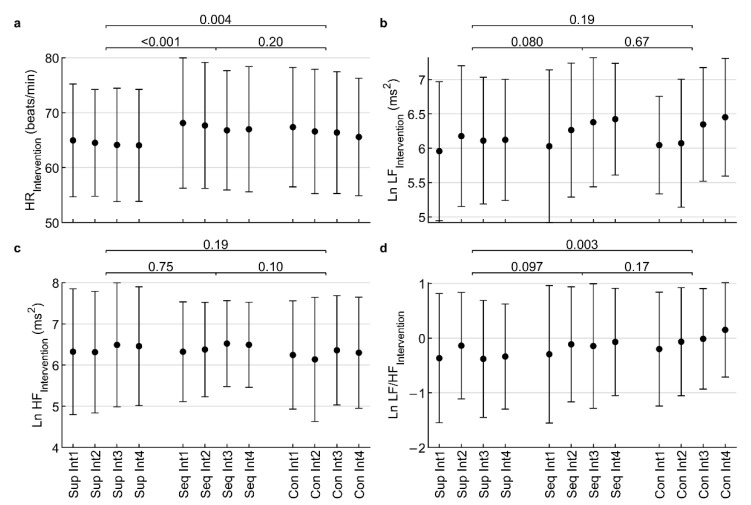
Influence of low-frequency sound on heart rate (**a**); low-frequency power (**b**); high-frequency power (**c**); and low-frequency/high-frequency ratio (**d**) during intervention lasting 4 × 5 min (Int1 to Int4). Ln—natural logarithm; Sup—placebo supine; Seq—stimulation with amplitude modulation of LFV; Con—constant LFV. The values displayed above the clips are *p*-values of ANOVA contrasts.

The Kolmogorov-Smirnov test showed that the indices Ln LF_Int_ (*p* = 0.24), Ln HF_Int_ (*p* = 0.38) and Ln LF/HF_Int_ (*p* = 0.13) did not differ significantly from the normal distribution. HR_Int_ differed significantly (*p* < 0.001) from the normal distribution, but after visual inspection of the histogram, deviations from normality were found to be small. ANOVA was considered robust enough for such deviations from normality [26]. For this reason, ANOVA could be used, despite it requiring the assumption of data normality. The results of the ANOVA are shown in Table 2 and Figure 5.

The interaction was not significant for any index (all *p* ≥ 0.76). The time factor was only significant for Ln LF_Int_ (*p* = 0.041). The intervention factor was significant for HR_Int_ (*p* < 0.001) and Ln LF/HF_Int_ (*p* = 0.011), so a more detailed analysis was performed using ANOVA contrast. The *Seq* intervention significantly (*p* < 0.001) increased HR_Int_ compared to placebo (Seq: 67.4 ± 11.4 beats/min, Pla: 64.4 ± 10.1 beats/min). The *Con* intervention significantly (*p* = 0.004) increased HR_Int_ compared to placebo (Con: 66.5 ± 11.0 beats/min, Pla: 64.4 ± 10.1 beats/min). The difference between the *Seq* and *Con* interventions was not significant (*p* = 0.20). In addition, the *Con* intervention significantly (*p* = 0.003) increased Ln LF/HF_Int_ compared to the placebo group (Con: −0.0 ± 1.0, Pla: −0.3 ± 1.0). However, the *Seq* intervention did not significantly (*p* = 0.097) change Ln LF/HF_Int_ compared to the placebo group (Seq: −0.2 ± 1.1, Pla: −0.3 ± 1.0). The difference between *Seq* and *Con* interventions was not significant (*p* = 0.17).

Thus, it can be said that LFS has a stimulating effect, because it increased the HR_Int_ caused by transfer of the activity of cardiac autonomous regulation from the vagus side to the sympathetic side.

### 3.3. The Effect of Low-Frequency Sound on the Subjective Perception of Stress and Mood

**Figure 6:**
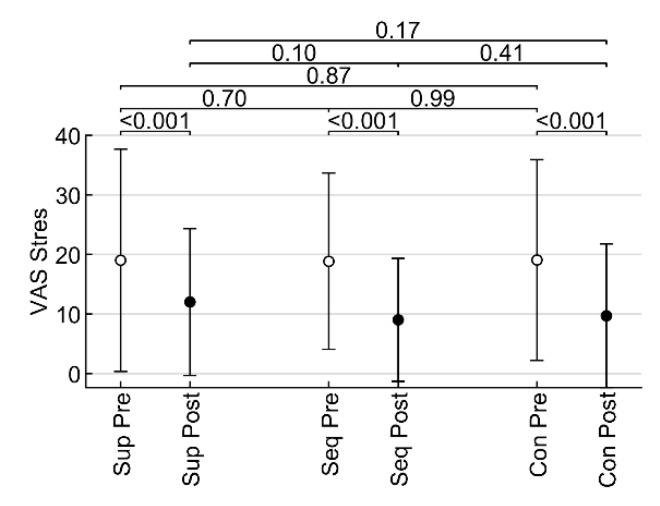
Influence of low-frequency sound on stress assessed by means of visual analogue scale in pre/post measures. Sup—placebo supine; Seq—sequentially dosed amplitude; Con—continuous amplitude. The values displayed above the clips are *p*-values of Fisher’s LSD tests.

Subjective perception of acute stress pre- and post-intervention was measured by VAS-stress. Comparing the values obtained from individual interventions, it is possible to infer a significant reduction in stress, which, however, occurred in all cases, including the intervention with sounds of nature (all *p* < 0.001). However, the differences between the values after the intervention were not statistically significant (all *p* ≥ 0.10) (Figure 6).

**Figure 7:**
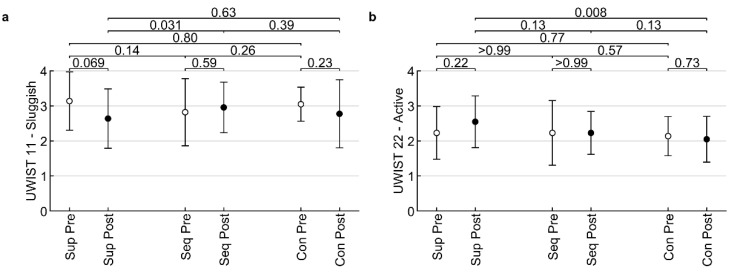
Influence of low-frequency sound on two items of UWIST – Sluggish (**a**) and Active (**b**) in pre/post measures. Sup—placebo supine; Seq—stimulation with amplitude modulation of LFV; Con –constant LFV. The values displayed above the clips are *p*-values of Fisher’s LSD tests.

Considering the effect of VAT on mood change, there were only two items from the UWIST questionnaire with significant differences (Figure 7). In the Slowed down item, there was a significant increase between the sequence amplitude and sounds of nature values (*p* = 0.031), while in the Active item there was a statistically significant decrease in constant amplitude values compared to the sounds of nature (*p* = 0.008). No significant changes were found for the other items (all *p* > 0.05).

## 4. Discussion

After all types of interventions, significant differences were measured in HRV (time factor) and there was a slight reduction in subjective stress perception (measured using VAS). However, when comparing the interventions, there was only minimal difference between the effect of constant LFV/amplitude modulation of LFV and sounds of nature, so this effect must be attributed mostly to factors other than LFV. It is possible that in all cases the recording with sounds of nature had an effect, but it could also be the fact that the participants lay still during all three conditions. In this case, we would need to find out if there would be a difference between a group that listened to sounds of nature when lying down and a group that did not listen to sounds of nature whilst lying down. 

These results differ from most previously published findings on the effect of VAT on physiological functions [11,12,13,14,15,19,20,21]. However, in almost all previous studies, LFV stimulation has been used in combination with music. Veternik et al. [34], however, applied only LFV to investigate its impact on the autonomic nervous system function and concluded that LFV did not have an effect. We could assume that physiological functions are influenced more by music than LFS. 

Another finding of this study is the effect of constant LFV on the sympathetic nervous system during the intervention itself (this effect could not be detected during the orthoclinostatic maneuver). This finding could be explained by empirical evidence of VAT practitioners, who have noticed that longer stimulation by constant LFV may provoke physiological irritation and worsen subjective feeling [10,16]. Similarly, we can explain why constant LFV had a significant effect on mood decrease in the item Activity (one of the items of UWIST-MACL), compared to nature sounds, and LFV amplitude modulation had a significant effect on the increase in the Slowed down item (again an item in UWIST-MACL). In this way, participants reflected stimulation of their sympathetic nervous system by feeling more active (as a result of constant LFV stimulation), whereas increase of parasympathetic activity was connected to feeling slowed down and probably more relaxed (as a result of amplitude modulation of LFV). Thus, we found differences between constant LFV and amplitude modulation of LFV. However, this study did not confirm the assumptions of significantly greater amplitude modulation of LFV efficiency on the autonomic nervous system. In particular, the expected significant effect on the parasympathetic nervous system was not observed.

Results of this study may be important for VAT practitioners and music therapists. It confirms (at least partially) the initial assumption that the effectiveness of VAT is increased by a combination of two effective stimuli, namely music and LFV [4]. However, the effectiveness of LFV (without music) on the autonomic nervous system and subjective stress perception has not yet been clearly demonstrated. Music was found to be a very strong stimulus for cardiac activity, and subjective perception and had a complex effect on nerve centres [35]. Even when combined with LFV, music probably remained the primary signal that determined ANS behaviour, whereas the application of LFV alone acted on the ANS differently. This could explain the inconsistent impact of LFV on the autonomic nervous system observed in studies that used LFV in combination with or without music. Moreover, LFV without music also only had a small effect on emotions. We do not have (as is the case with music [35]) culturally imprinted patterns for the perception of LFV, and, therefore, the effect of LFV on mood is probably much more unpredictable. 

Recommendations for future research:

- To further investigate the effect of LFV on the autonomic nervous system, we recommend conducting an experimental study that would compare the effect of music versus LFV with music (or also only LFV without music) measured by HRV or similar outcome measures, to find out if the effect of LFV is influenced by various accompanying stimuli.
- We recommend that the specific part of the autonomic nervous system also be considered in future studies, as it is possible that the vagal response to LFV stimulation differs from the sympathetic response.
- In this research experiment, the time span between the interventions was one week. Due to the fact that no statistically significant differences were found in the VSF values measured in the standing and lying positions after the intervention, the LFV effect seems to persist only for a short time after the end of the stimulus and is relatively quickly masked by environmental disturbances. Thus, the intervals between interventions could be only one day in future research studies.
- We recommend to only recruit students with high levels of stress in future studies focused on the research of stress.
- The use of VAT can be further explored in patients suffering from multiple diseases.

### Strengths and Limits of the Study

Vibroacoustic research requires further studies to verify the effectiveness of LFV, especially through physiological measurements. A small number of studies with HRV have been realized so far. The differences between the effects of LFV without music and with music have not been sufficiently explored, and, above all, research into various LFV parameters, such as frequencies or amplitude modulations, is completely lacking. Although this study did not prove the expected effect of LFV, it allows space for hypotheses that need to be explored by other research studies (mainly in clinical areas) and that may be essential to understand the effect of LFV administered with and without music. 

We tried to adhere to relatively strict criteria for standardization of measurements and a relatively homogeneous sample. Nevertheless, there were some pre-test differences in participants, which could be caused by weekly delays between measurements or factors that we did not detect, such as spontaneous physical activity. For similar studies in the future, we propose to shorten the time between measurements; optimally, to perform measurements in a sequence of consecutive days.

The blinding procedure also showed certain limits given by the inter-subject design of the study. Blinding could only be effective in relation to the impossibility of identifying amplitude modulations, not in relation to control measurements (listening to sounds of nature). Constant LFV and LFV with amplitude modulation were generated on two different types of vibroacoustic beds, which do not have the same technical solutions. In future studies it would be more appropriate to conduct research on only one vibroacoustic bed. 

The relatively small number of participants was also problematic, but the course of the study was interrupted by COVID-19 and, due to pandemic restrictions, it was not possible to continue the research and obtain a larger data set. Furthermore, this study was designed as a pilot study in preparation for a more robust RCT that is already registered at ClinicalTrial.gov (NCT04293848). We decided to use Vibrobed for this RCT study because we would like to research the effect of amplitude modulations and other LFV characteristics.

In addition, the participants did not have a sufficiently high level of stress before the experiment, and it is possible that the differences in interventions could not be sufficiently manifested for this reason. In this study we did not only recruit students with high stress levels, because we expected much stronger effects from the intervention. We realise this could have had a negative impact on the data and we recommend recruiting only students with higher levels of stress in future studies. Before the intervention, the participants (due to HRV measurements) lay for 7 min, a time that could serve to ensure sufficient relaxation of the participant before the intervention and to influence the data obtained during successive measurements. Although research in clinical populations, such as those with high stress levelsthat affect the activity of the autonomic nervous system, is more demanding, it could yield more favourable results in verifying the effectiveness of VAT. In addition, some of the measurement tools used were probably not sensitive enough to capture the changes caused by the interventions, and in the case of HRV, its sensitivity to several disturbing factors may also be problematic.

Last, but not least, the study protocol was not registered for this study (considering its pilot character), although there was no significant deviation from the pre-established experimental design during the study.

## Figures and Tables

**Table 1:** Influence of low-frequency sound on heart rate variability indices in pre/post-test measures.

Variable	Time	Intervention	Interaction
	*p*	*p*	*p*
HR_Standing_ (beats/min)	0.010	0.21	0.76
HR_Supine_ (beats/min)	<0.001	0.054	0.68
Ln LF_Standing_ (ms^2^)	<0.001	0.11	0.28
Ln LF_Supine_ (ms^2^)	0.072	0.63	0.90
Ln HF_Standing_ (ms^2^)	<0.001	0.12	0.99
Ln HF_Supine_ (ms^2^)	0.019	0.88	0.85
Ln LF/HF_Standing_	<0.001	0.31	0.076
Ln LF/HF_Supine_	0.57	0.80	0.98

*p*—significance of ANOVA factors; HR—heart rate; Standing—standing body position in orthoclinostatic maneuver; Supine—supine body position in orthoclinostatic maneuver; Ln—natural logarithm; LF—low-frequency power; HF—high-frequency power; LF/HF = LF/HF ratio.

**Table 2:** Influence of low-frequency sound on heart rate variability indices during intervention.

Variable	Time	Intervention	Interaction
	*p*	*p*	*p*
HR_Int_ (beats/min)	0.42	<0.001	>0.99
Ln LF_Int_ (ms^2^)	0.041	0.19	0.84
Ln HF_Int_ (ms^2^)	0.35	0.23	>0.99
Ln LF/HF_Int_	0.20	0.011	0.76

*p*—significance of ANOVA factors; HR—heart rate; Int—intervention; Ln—natural logarithm; LF—low-frequency power; HF—high-frequency power; LF/HF = LF/HF ratio.

## Data Availability

The data presented in this study are available on request from the corresponding author.

## Appendix A. Informed Consent

**INFORMED CONSENT**
For research project: VAS (project IGA) Implementation period: 4-2019/2-2020 project leader: doc. Mgr. Jiří Kantor, Ph.D. Madam, sir, We are writing to you to ask you to collaborate on a research project involving an experiment involving listening to sounds and taking measurements before, during and after the experiment using the methods listed below. All procedures used during the experiment and measurements are non-invasive and safe, except for the risks listed below. Only nature sounds and low frequency sounds will be used in the experiment. **The purpose of the study** is to investigate the effect of the sounds used in the experiment on the human body. **Measurement methods:** The composition of data collection methods includes non-invasive instrumental and psychological test instruments: - An initial questionnaire to obtain personal and demographic data, such as age, gender, incidence of health problems, etc. and other specific questions related to current psychosomatic status. - Analysis of heart rate variability using frequency and time indices. - Blood pressure and respiratory rate measurements. - Visual analogue scales to detect differences in subjective perception of stress levels. - UWIST Mood Adjective Checklist to determine the difference in emotional tuning before and after the intervention. - The standardised Perceived Stress Scale questionnaire (Cohen, 1983) in the Czech version called the “Škály vnímaného stresu”, as presented by Buršíková Brabcová and Kohout (2018). - Interview to supplement data of a rather qualitative nature. **Time Schedule:** The total length of one session is 60 min and will take place once a week, always on the same day and time. There will be a total of three sessions, so the length of participation in the research is three weeks. The measurements will take place at two university departments, PdF and FTK. The results of the research will be shared after the study is completed. The **following risks** are known for the intervention implemented in the research experiment: - Hypotonia - Angina pectoris - Psychotic states - Post-traumatic conditions - Open bleeding The participant of the study acknowledges by his/her signature (below) that he/she does not suffer from any of the above mentioned risks at the time of the experiment. **The participant of the study agrees:** - To avoid intake of addictive substances in the morning hours before the experiment (drinks containing caffeine and tannin, cigarettes) and strong addictive substances the day before the experiment (alcohol, marijuana). - With taking heart rate recordings, blood pressure measurements and audio recording of the qualitative interview. - With anonymous direct quotation of the data from the interview. - With the retention of all research records, which will be used anonymously only for the purposes of the research study. **Statement** I declare that I agree to participate in the project mentioned above. I have been informed by the project leader about the nature of the research and have been informed about the objectives and the methods and procedures that will be used in the research, as well as the benefits and risks for me of participating in the project. I agree that all data collected will be used for research purposes only and that the results of the research may be published anonymously. I have had the opportunity to consider everything properly, calmly and in sufficient time. I have had the opportunity to ask the researcher everything I considered essential and necessary to know. I received a clear and understandable answer to these questions. I am informed that I have the possibility to withdraw from the project at any time, even without giving a reason. The researcher declares that personal information that could identify the research participant will not be passed on to anyone or appear in any publication outputs. Personal data will be protected in accordance with applicable legislation. This informed consent is made in two copies, each with the validity of an original, one of which will be given to me (or my legal representative) and the other to the project investigator. Name, surname and signature of the project leader: ____________________________________________________________ In______________________ on:_________________________________ Name, surname and signature of the participant in the project: ____________________________________________________________ In______________________ on:_________________________________

## Appendix B. Protocol for the Collection of Demographic/Personal Data

**ENTRY PROTOCOL—VAS 2019**

**Research participant code:**

Group number/Participant code/Meeting number: …………/………………/………….

This anonymous data is only filled in before the research begins. Thank you for your time and willingness to complete the survey.

**1. Gender**

a. Male

b. Female

**2. Age..............**

**3. I have a somatic/other medical disability or long-term/acute illness** that is usually considered more serious:

a. no

b. yes

If yes, please specify which ones:

.......................................................................................................................................................

**4. Regular medication?**

a. no

b. yes

If yes, please state what medication you are taking and for what:

.......................................................................................................................................................

**5. Menstrual cycle (women only)—**follicular (1–14 days) and luteal (14–28 days) phases.

Please indicate the start of your menstrual cycle:...........................or what phase of your cycle you are in:.............................

**6. Sleep quality—**regular and sufficient sleep (at least 6–7 h)?

a. yes

b. no

**7. I am not currently receiving psychiatric treatment.**

a. yes

b. no

If you are receiving psychiatric treatment, please indicate the diagnosis or reason for treatment (voluntary):

.......................................................................................................................................................

*If there is a mental disorder that could affect the research measurements, consultation with the supervision team is required.*

Date:

Signature of the researcher:

## Appendix C. Visualisation of Amplitude Modulation Used by Vibrobed

**Table A1:** Parameters of audio-recording for Vibrobed.

	AM 1 (14 s)		AM 2 (10 s)		AM 3 (6 s)		Calibration 40 Hz	
Zone (Vibrobed)	dBmax	Pmax (W)	dBmax	Pmax (W)	dBmax	Pmax (W)	−15 dB RMS	W
Z1	−9.15	0.89	−9	0.92	−10.1	0.71	0.68	0.23
Z2	−9.15	0.86	−9	0.89	−10.1	0.69	0.67	0.22
Z3	−9.15	1.02	−9	1.06	−10.1	0.82	0.73	0.27
Z4	−9.15	1.02	−9	1.06	−10.1	0.82	0.73	0.27

Z1—calves area; Z2—thighs area; Z3—low back area; Z4—upper back area; Pmax (W)—Nominal Power (Watt); dBmax—Maximum decibel level; RMS (Root mean squer)—Average transducers power.
